# Mirroring everyday clinical practice in clinical trial design: a new concept to improve the external validity of randomized double-blind placebo-controlled trials in the pharmacological treatment of major depression

**DOI:** 10.1186/1741-7015-10-67

**Published:** 2012-07-02

**Authors:** Emanuel Severus, Florian Seemüller, Michael Berger, Sandra Dittmann, Michael Obermeier, Andrea Pfennig, Michael Riedel, Sophia Frangou, Hans-Jürgen Möller, Michael Bauer

**Affiliations:** 1 Department of Psychiatry and Psychotherapy, University Hospital Carl Gustav Carus, Technical University Dresden, Fetscherstr. 74, 01307 Dresden, Germany; 2 Department of Psychiatry and Psychotherapy, LMU Munich, Nussbaumstr. 7, 80336 Munich, Germany; 3 Vinzenz von Paul Hospital, Schwenninger Str. 55, 78628 Rottweil, Germany; 4 Institute of Psychiatry, King's College London, De Crespigny Park, London SE5 8AF, UK

**Keywords:** major depression, clinical trials, randomized controlled trials, psychopharmacology

## Abstract

**Background:**

Randomized, double-blind, placebo-controlled trials constitute the gold standard in clinical research when testing the efficacy of new psychopharmacological interventions in the treatment of major depression. However, the blinded use of placebo has been found to influence clinical trial outcomes and may bias patient selection.

**Discussion:**

To improve clinical trial design in major depression so as to reflect clinical practice more closely we propose to present patients with a balanced view of the benefits of study participation irrespective of their assignment to placebo or active treatment. In addition every participant should be given the option to finally receive the active medication. A research agenda is outlined to evaluate the impact of the proposed changes on the efficacy of the drug to be evaluated and on the demographic and clinical characteristics of the enrollment fraction with regard to its representativeness of the eligible population.

**Summary:**

We propose a list of measures to be taken to improve the external validity of double-blind, placebo-controlled trials in major depression. The recommended changes to clinical trial design may also be relevant for other psychiatric as well as medical disorders in which expectations regarding treatment outcome may affect the outcome itself.

## Background

The treatment of patients with major depression remains one of the major challenges in psychiatry in the 21st century [1-3]. There is a general consensus that antidepressant medications are an essential component of the treatment plan at any stage of the illness and particularly in severe cases [4,5].

In the era of evidence-based medicine, randomized, double-blind, placebo-controlled, trials constitute the gold standard for testing the efficacy of new pharmacological interventions for major depression [6,7]. Their unique strength derives from the fact that they allow comparisons of a psychopharmacologically active medicine to an identical looking 'medicine' which does not contain any active ingredient (placebo). Randomization is thought to ensure unbiased allocation of patients to each treatment arm while blinding both patients and investigators to treatment allocation safeguards against biased reporting of potential benefits or adverse effects [8].

During the last few years however it has become increasingly evident that the very strength of this type of trial, that is, the blinded use of placebo, may itself influence antidepressant and placebo response and remission rates [9,10] and consequently clinical trial outcome [11]. As neither randomization nor double-blinded placebo administration are part of normal clinical practice the external validity of results obtained from randomized, double-blind, placebo-controlled trials may not represent real life outcomes [12,13]. Some of these limitations are introduced by the stringent eligibility criteria of clinical trials that routinely exclude patients with complex presentations [14,15]. In addition, the acceptability of eligible patients to be randomized to the placebo arm is considered to be highly variable thus further limiting the number of participants to enroll in such studies [16]. In many cases differences in outcome-relevant demographic and clinical characteristics of eligible patients that were enrolled (enrollment fraction) versus those that refused are not described in detail, which makes it difficult to draw reliable conclusions regarding the external validity of the trial [17]. Therefore, a new study design is needed which reconciles the needs of clinical research meeting highest methodological standards (double-blinding, placebo-control) and at the same time preserves the external validity of the findings derived from these studies [18].

## Discussion

While clinical trials in major depression focus on the antidepressant efficacy of medications, clinical care has a wider remit and is concerned with the overall treatment of individual patients suffering from this disorder. In the context of clinical care the medication offered is already approved and the choice of the specific antidepressant does not happen in a randomized fashion but through a process of shared decision making between the treating physician (primary care doctor, psychiatrist) and the patient [19-21]. During this process patients are informed of the potential consequences of treatment choices including expected benefits. The combination of using an approved antidepressant and the patients' active participation in treatment choices creates positive expectations regarding the effectiveness of the treatment agreed upon and may positively affect the overall outcome [22]. In contrast, in any randomized double-blind placebo-controlled trial, and particularly in Phase III trials, the expectation of a positive outcome is lessened by the knowledge that participants may be randomized to the placebo arm [9-11,23-26]. However, in the absence of alternatives, only those drugs proven efficacious in the aforementioned double-blind, placebo-controlled randomized trials have been approved for use for the treatment of major depression. To improve external validity of randomized, double-blind, placebo-controlled trials in major depression we propose to introduce two core features of everyday clinical practice, namely shared decision making and availability of the active medication to all participants, into randomized, double-blind, placebo-controlled trials and to study the effects of this intervention on study outcome and recruitment.

### Mirroring clinical practice in double-blind, placebo-controlled trials

The introduction of the core features outlined above will be achieved in a number of steps:

1) Active medication for all study participants: At the end of late phase III clinical trials study participants could be offered the option of receiving the active medication or to remain on the study medication if it had been efficacious and well tolerated. The duration of this non-randomized phase could vary according to clinical need and patient preference.

2) Detailed explanation of potential benefits: Placebo is often referred to as an 'inert and deceptive intervention intended to please or placate the patient but without any potential to produce meaningful therapeutic results' [27]. While not explicitly stated in the informed consent form (ICF) patients may consequently conclude that only if they are assigned to the active medication will they have a reasonable chance to improve regarding their depression. As this is not true we propose to reconceptualize what a placebo - and being randomized to the placebo arm - constitutes. This would involve explaining to study participants that improvement in their depressive symptoms may occur spontaneously while on the placebo arm, in which case they may have been spared unnecessary medication [25]. Additionally it should be emphasized that study participation is likely to result in increased and more regular contact with clinicians and closer monitoring of their symptoms which can be beneficial in its own right [28].

The combination of these features mirrors clinical practice and redresses patients' fears of a negative outcome following study participation [22]. An appropriate tool to convey the above mentioned information seems to be the informed consent form [29]. Traditional informed consent forms currently state that 'A placebo or inactive medicine looks like real medicine but it is not. It is a dummy or pretend medicine. It has no effect on a person because it has no real medicine in it' (World Health Organization Research Ethics Review Committee). In contrast to this statement we propose the following (or similar) wording for a double-blind placebo-controlled randomized trial in major depression:

'The goal of this trial is to find out whether patients receiving drug 'X' will have an additional benefit compared to those receiving a placebo. A placebo or inactive medicine looks like real medicine, but contrary to a real medicine, it does not contain any substance believed to influence brain mechanisms relevant to depression. However, patients receiving placebo in clinical trials for depression often report significant improvements in their symptoms. There may be several reasons for this. Firstly, depressive symptoms are known to improve even without treatment as part of the natural course of the illness. Second, having frequent and regular discussion about your feelings and progress with the study clinicians may be therapeutic in itself. Third, being positive about getting better during the clinical trial may actually contribute to improving your symptoms [22,30]. Therefore, regardless of the type of medicine you might receive (X or placebo) you may benefit from participating in this trial. Finally, at the end of the study you will be offered the option of receiving the active medication or remaining on the study medication. You can then decide together with your doctor what would be the appropriate next steps. If the study medication had been of benefit to you, you may want to continue on this medicine. In contrast, if you and your study doctor feel that you had not responded satisfactorily to the study medication, you may want to vote for the option of being given X if clinically appropriate'.

### Research agenda

In order to test the impact of shared decision making and of study drug availability to all participants we propose to start with a number of Phase IV trials. In this agenda we will refer to a study design incorporating these proposed changes as an 'enhanced randomized controlled trial (RCT)'. This agenda should focus on comparing the antidepressant efficacy of study drug 'X' when evaluated using a traditional double-blind RCT versus the proposed enhanced RCT design. The evaluation will consist of comparisons of the effect size of placebo-active treatment arms differences on depressive symptoms and on outcome-relevant demographic and clinical characteristics of enrolled patients. This would then allow an estimation of the representativeness of the enrolled patients compared to all patients in the eligible target patient population.

The set of trials we propose to conduct consists of two steps:

In the first step potentially eligible patients suffering from a major depressive episode in the context of a major depressive disorder will be asked about their willingness, in general, to participate in clinical trials to evaluate different study designs on antidepressant efficacy. They will be given a first informed consent form (ICF#1) which will outline the general purpose of the trial but will not include details regarding study design. If patients are interested they will be asked to sign ICF#1. By doing so they give permission to study clinicians to access and analyze outcome relevant demographic and illness-related variables and check their eligibility for inclusion in clinical trials. This may also involve drawing blood for laboratory tests. Those signing ICF#1, and who fulfill eligibility criteria, will then be randomized to one of the following two treatment trial set ups (not treatment arms):

1) traditional double-blind, randomized, placebo-controlled eight-week trial, including a traditional informed consent form, no option for extension period; or

2) enhanced double-blind, randomized, placebo-controlled eight-week trial, including the novel informed consent form outlined above, option for extension period.

The two treatment trials will be conducted by independent research teams who will be unaware that the two trial designs will be compared. Each clinical trial team will approach patients randomized to their study design (traditional or enhanced) and will ask them for their consent to participate. The wording of the ICF#2 will differ between the two trial designs. Patients approached to participate in the traditional trial will be presented with the traditional version of the ICF#2 while patients approached to participate in the enhanced trial will be given the new proposed ICF#2 outlined above.

At study completion the traditional and enhanced designs will be compared in terms of:

1) Efficacy, by comparing the outcomes of the two trial designs based on the effect size symptom score improvement, number of responders, number of patients in remission, number of patients withdrawing, and Number Needed to Treat;

2) Tolerability, by comparing the outcomes of the two trial designs based on number and type of reported adverse events; and

3) Representativeness of study participants, by comparing outcome-relevant demographic and clinical characteristics of enrolled patients in each design (enrollment fraction) to the respective eligible target clinical population.

Comparisons between the two study designs can be straightforward and performed using ordinary linear models with treatment success being explained by study arm, study design and their interaction. As the goal is to assess study designs rather than specific drugs, the active medication to begin with, in both the traditional and enhanced clinical trials outlined, will be sertraline which has already been approved for the treatment of depressive disorder and is thought to have a balanced profile of efficacy and tolerability [31,32]. For the purpose of this project, the research teams on both clinical trial designs will be blind to the active drug so as not to influence their evaluation. Equally patients will be told in the ICF#2 that they will be randomized 'either to placebo or to a drug thought to be helpful in depression'. In addition precise information will be provided about potential side effects which will be reassuring to all study participants. In this way we will be able to use existing literature on sertraline for power calculations for the traditional and enhanced clinical trials examined here. Additionally, this availability of multiple previous sources of information about sertraline will allow us to discuss findings from this project in the context of the wider literature.

## Summary

In the era of evidence-based medicine it is paramount that we produce clinically relevant scientific evidence for the pharmacological treatment of patients with major depression. We therefore propose to enhance double-blind, placebo-controlled trials in such a way as to reflect clinical practice more closely. This could be achieved by adding extensions, thus offering every patient the possibility of receiving active treatment and formally integrating the process of shared decision making while obtaining informed consent. The proposed changes in the process of informed consent focus on a balanced presentation of evidence regarding the benefit of study participation irrespective of whether patients are assigned to active or placebo treatment. In order to evaluate the impact of the study designs on the efficacy and enrollment fraction of an intervention we further outlined a research agenda involving direct comparison of traditional and enhanced trial designs. While we feel that our approach represents an important step towards a more comprehensive assessment of the efficacy and external validity of the treatment options available, further research is needed in this area to improve the clinical care of patients suffering from this complex and often severely debilitating psychiatric illness [33]. Finally, while our proposal primarily targets major depression its central idea may also be relevant for other psychiatric disorders as well as medical disorders such as asthma, in which expectations regarding treatment outcome may affect the outcome itself [34,35].

## List of abbreviations

ICF: informed consent form; RCT: randomized controlled trial.

## Competing interests

Emanuel Severus has been on the speakership bureaus of AstraZeneca, Eli Lilly and Company and Lundbeck. Florian Seemüller received grants and research support from Lilly, Astra Zeneca and Glaxo-Smith Kline. He received honoraria from Lundbeck, Bristol-Meyers Squibb, Lilly and Astra Zeneca, Bial, BMS, Cephalon, Eli Lilly, Glaxo-Smith Kline, Janssen-Cilag, Organon, Pfizer Inc, Sanofi -Aventis, Servier, UBC and UCB Belgium. Andrea Pfennig received a stipend/research support from GlaxoSmithKline and research support from AstraZeneca. She has received speaker honoraria from AstraZeneca and Eli Lilly and Company. Michael Riedel has received research grants/support or has served as a consultant for AstraZeneca, Pfizer, Otsuka Pharma, and Janssen-Cilag. In the context of investigator-initiated trials, M. Riedel has received support from AstraZeneca and Pfizer.

Sophia Frangou has served as a consultant on advisory boards for Janssen Cilag and Bristol-Myers Squibb/Otsuka. Hans-Jürgen Möller has received grants or is a consultant for and on the speakership bureaus of AstraZeneca, Bristol-Myers Squibb, Eisai, Eli Lilly, GlaxoSmithKline, Janssen Cilag, Lundbeck, Merck, Novartis, Organon, Pfizer, Sanofi-Aventis, Schering-Plough, Schwabe, Sepracor, Servier and Wyeth. Michael Bauer has received Grant/Research Support from The Stanley Medical Research Institute, NARSAD and the European Commission (FP7). He was/is a consultant for AstraZeneca, Lilly, Servier, Lundbeck, Bristol-Myers Squibb and Otsuka. Michael Bauer has received Speaker Honoraria from AstraZeneca, Lilly, GlaxoSmithKline, Lundbeck, Bristol-Myers Squibb and Otsuka. Michael Berger, Sandra Dittmann and Michael Obermeier have no conflicts of interest to declare.

## Authors' contributions

All authors have made substantive intellectual contributions to the paper. ES conceived of the paper and drafted the manuscript. FS assisted in conceiving of the paper and helped to draft the manuscript. MBerger, SD, MO, MR, SF, HJM and MBauer all helped to draft the manuscript. All authors read and approved the final manuscript.

## Pre-publication history

The pre-publication history for this paper can be accessed here:

http://www.biomedcentral.com/1741-7015/10/67/prepub
